# The effect of different implant‐abutment types and heights on screw loosening in cases with increased crown height space

**DOI:** 10.1002/cre2.894

**Published:** 2024-06-16

**Authors:** Amirreza Hendi, Sobhan Mirzaee, Mehran Falahchai

**Affiliations:** ^1^ Department of Prosthodontics, School of Dentistry, Dental Sciences Research Center Guilan University of Medical Sciences Rasht Iran; ^2^ School of Dentistry, Dental Sciences Research Center Guilan University of Medical Sciences Rasht Iran

**Keywords:** dental abutments, dental implants, dental implant loading

## Abstract

**Objectives:**

The stability of the abutment screw is pivotal for successful implant‐supported restorations, yet screw loosening remains a common complication, leading to compromised function and potential implant failure. This study aims to evaluate the effect of different implant‐abutment types and heights on screw loosening in cases with increased crown height space (CHS).

**Materials and Methods:**

In this in vitro study, a total of 64 abutments in eight distinct groups based on their type and height were evaluated. These groups included stock, cast, and milled abutments with heights of 4 mm (groups S4, C4, and M4), 7 mm (groups S7, C7, and M7), and 10 mm (groups C10 and M10). Removal torque loss (RTL) was assessed both before and after subjecting the abutments to dynamic cyclic loading. Additionally, the differences between initial RTL and RTL following cyclic loading were analyzed for each group (*p* < .05).

**Results:**

The C10 group demonstrated the highest RTL, whereas the S4 group exhibited the lowest initial RTL percentage (*p* < .05). Furthermore, the study established significant variations in RTL percentages and the discrepancies between initial and postcyclic loading RTL across different abutment groups (*p* < .05). Additionally, both abutment types and heights were found to significantly influence the RTL percentage (*p* < .05).

**Conclusion:**

The type and height of the implant abutment affected screw loosening, and in an increased CHS of 12 mm, using a stock abutment with a postheight of 4 mm can be effective in minimizing screw loosening.

## 1 INTRODUCTION

The long‐term success and stability of implant‐supported restorations depend on various factors, including preventing screw loosening (Seddigh & Mostafavi, 2019). Screw loosening can compromise the stability and integrity of the implant restoration, leading to functional and aesthetic issues for the patient (Huang & Wang, 2019; Kourtis et al., 2017). Implant abutments need to be crafted using biocompatible materials with suitable mechanical properties to meet the biological, functional, and aesthetic requirements (Saini, 2015). Moreover, implants should exhibit precise and passive fitting onto the corresponding implants to minimize potential complications, including screw loosening, bone loss, and abutment fractures during functional usage (Graf et al., 2023; Omori et al., 2020).

To achieve optimal mucogingival aesthetics, implant abutments should also exhibit an appropriate emergence profile, essential for supporting the surrounding soft tissue (de Freitas et al., 2023). Screw loosening is a common complication in implant‐supported restorations, impacting stability and necessitating preventive measures (Kourtis et al., 2017). The abutment screw loosening in the first year after loading was reported to be more than 5%, which increases by the year (Lee et al., 2020). The success of implant treatment requires a dynamic balance between biological (such as inflammation) and biomechanical factors (Gao et al., 2019). Encompassing inadequate screw tightening force, settling phenomenon, vibrational micro‐movements, excessive bending, mismatch between prosthesis and abutment or abutment and implant, design and dimensions of the restoration, biomechanical overload, suboptimal implant positioning, augmented off‐axis forces, and extended cantilever length have been reported to associate with abutment screw loosening (Londhe et al., 2020).

Crown height space (CHS) refers to the vertical distance between the bone crest and the occlusal plane. In the context of fixed implant prostheses, an ideal CHS measurement is 8−12 mm (Bagegni et al., 2021). However, in cases where vertical space has not been corrected through bone grafting, longer abutments may be necessary. The distribution of CHS in implant abutments occurs between the collar portion and the segment of the abutment covered by the crown (Bordin et al., 2019). Longer abutments function as force amplifiers, mainly when there is an increase in vertical cantilever (Sun et al., 2023). Due to its lever‐arm effect, the vertical cantilever leads to an augmentation of the forces exerted on the abutment screw (Gehrke, 2015). The biomechanical aspects of CHS are closely associated with the lever‐arm mechanism.

Different abutment designs and heights can affect the distribution of forces and stresses within the implant system, potentially impacting the preload of the screw and its resistance to loosening (Shash et al., 2019). Dentists are confronted with diverse options when selecting an abutment, including stock, cast, milled (custom‐made using computer‐aided design/computer‐aided manufacturing (CAD/CAM) types, and so on (Vinhas et al., 2020). In critical situations of the treatment plan, dentists face the additional challenge of determining the appropriate abutment height and the choice of abutment type (Ramalho et al., 2020; Târtea et al., 2023).

The research conducted on abutment height's effect on biomechanical complications is mostly limited to one type of abutment and different CHS values (Elsayed et al., 2021; El‐Sheikh et al., 2018). Understanding the impact of abutment characteristics on screw loosening can provide valuable insights for clinicians in selecting the most suitable abutment design and height to minimize the risk of screw loosening and enhance the long‐term stability of implant restorations. In this regard, we aimed to investigate the effect of the type of direct implant abutments (stock, cast, and milled abutments) and their height on the initial loosening of the increased CHS.

## 2 METHODS

This in vitro study was confirmed by the ethical committee of the Guilan University of Medical Sciences [IR.GUMS.REC.1400.043]. To calculate the sample size, considering the statistical power of 0.80, the error level of 0.05, and the SD obtained from the study by Park et al. (Park et al., 2014) 3.05 (cast group) and 7.48 (CAD/CAM group), a minimum sample size of eight was obtained for each group.

The study involved eight groups (*n* = 8) categorized by the type and height of the abutment: stock abutments with a postheight of 4 mm (S4, Hex cemented abutment, Dio UF), cast abutments with a postheight of 4 mm (C4), milled abutments with a postheight of 4 mm (M4), stock abutments with a postheight of 7 mm (S7, Hex cemented abutment, Dio UF), cast abutments with a postheight of 7 mm (C7), milled abutments with a postheight of 7 mm (M7), cast abutments with a postheight of 10 mm (C10), and milled abutments with a postheight of 10 mm (M10). The abutment diameter and collar height were considered 5.5 and 2 mm, respectively.

**Figure 1:**
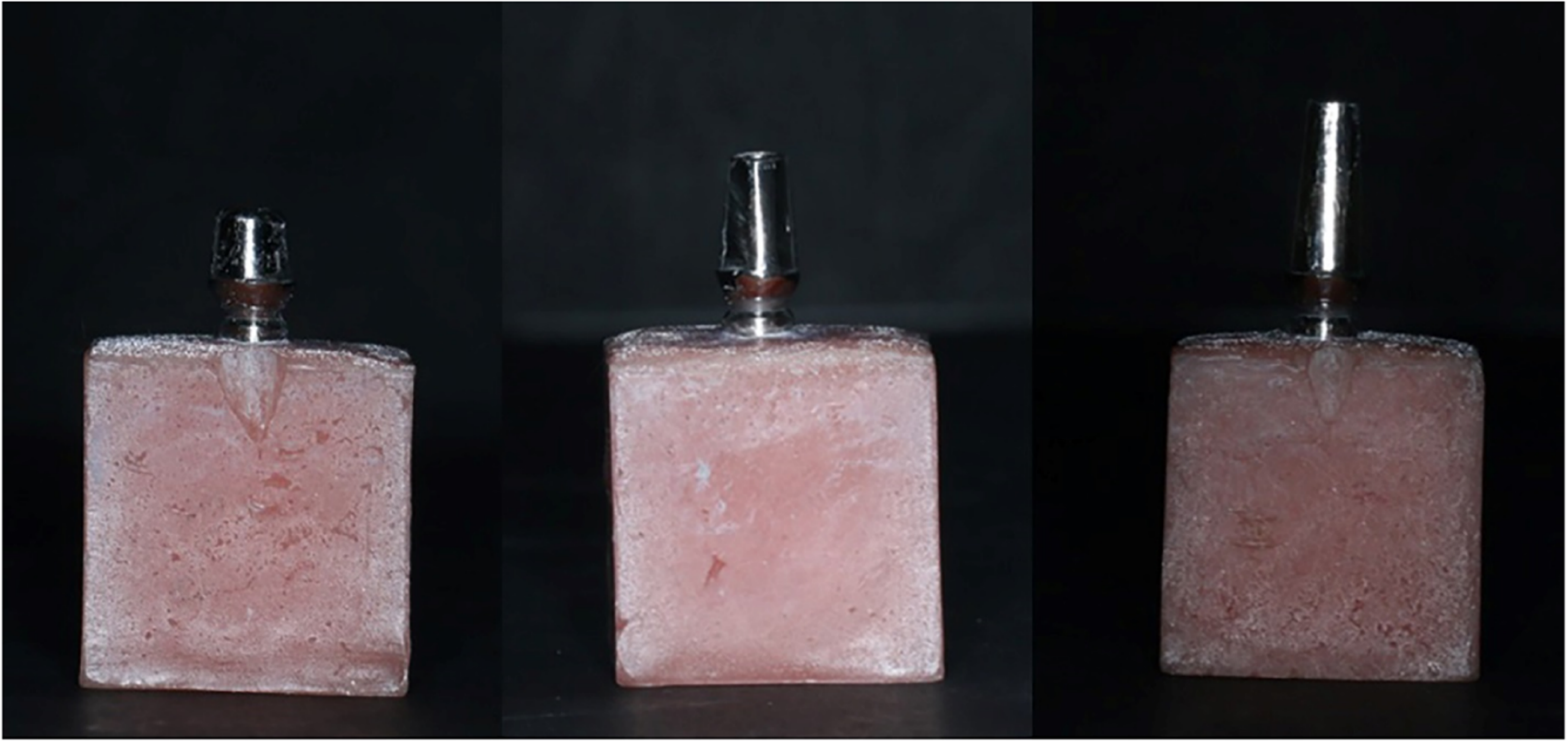
Milled abutment with postheights of 4, 7, and 8 mm.

**Figure 2:**
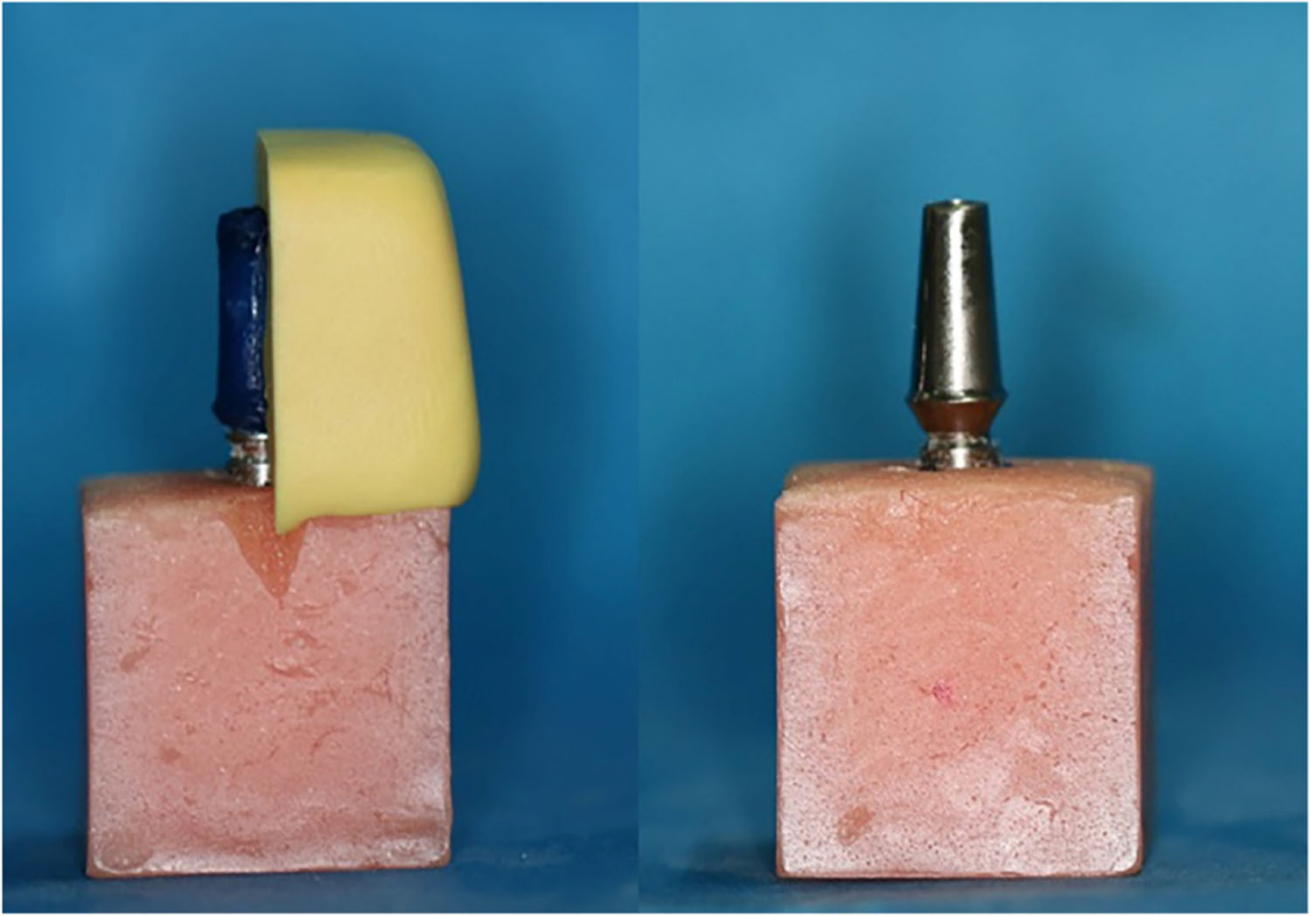
Fabrication of a cast abutment using a putty index.

In milled abutments groups, Titanium pre‐milled blanks (ARUM premilled blank, Doowon) were used to fabricate abutments with postheights of 4, 7, and 10 mm (Figure 1) using CAD/CAM system (ARUM 5X‐200, Doowon). To create casting abutments with postheights of 4, 7, and 10 mm, indices obtained from milled abutments were used. Wax patterns were formed based on them (Figure 2). Then, during the casting process, a Co‐Cr alloy (Wirobond C, Bego) was used.

All abutments were tightened on their respective analogs, and the assembly of abutment‐analog was positioned in the center of a wax block measuring 20 × 20 × 20 using a surveyor. Subsequently, the mold was filled with auto‐polymerizing acrylic resin (Acropars, Marlik, Tehran, Iran) up to 1 mm from the junction of the abutment and analog. Following mounting the abutments, all the screws were tightened according to the protocol of Siamos. Siamos et al. (2002), twice at 10‐min intervals using a torque meter (BTG60, Tohnichi, Tokyo, Japan) to a force of 30 Ncm, and the removal torque value (RTV) was measured after 5 min using a calibrated electronic torque meter with an accuracy of 0.1 Ncm (Lutron Electronic, Lutron Electronic Enterprise Co.). After measuring the RTV, the abutment screws were retightened to 30 Ncm.

The abutments were scanned using a laboratory scanner (Ceramill Map 600, Amann Girrbach) for designing ceramic crowns. The crown height was set to a maximum of 12 mm within the normal range. The occlusal surface of the crowns was made flat and perpendicular to the fixture‐abutment axis with a 10‐mm diameter for proper contact (El‐Sheikh et al., 2018). A 30‐µm space was allocated for luting cement. Zirconia blanks (Zolid fx white, Amann Girrbach) were milled using a milling machine (Ceramill Matik, Amann Girrbach), and the crowns were sintered in a furnace (Ceramill sintron, Amann Girrbach) at 1450°C for 9 h. Crowns were then luted to the abutments using Tempbond temporary cement (Kerr).

**Figure 3:**
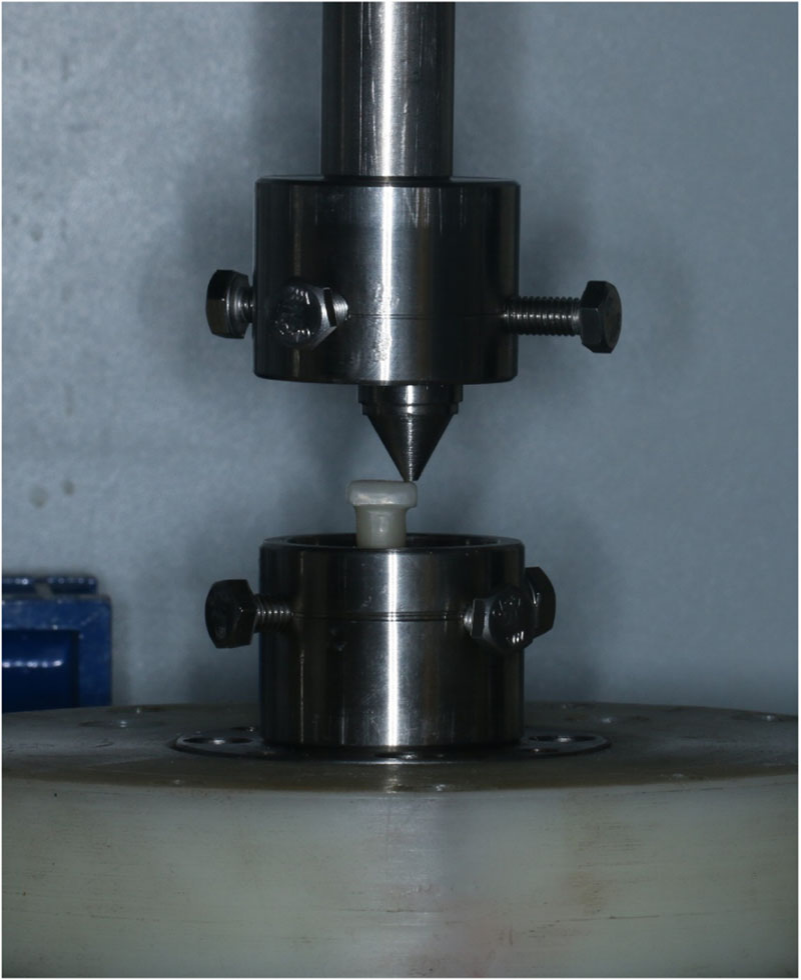
Application of cyclic loading.

In the next step, each sample was subjected to 500,000 cycles of 75 Ncm of force (equivalent to 6 months of adult human chewing) using a chewing simulator device (Chewing Simulator, S‐D Mechatronic) (Siadat et al., 2015). The movable piston of the device contacted the ceramic crown at a distance of 5 mm from the central axis of the fixture (Figure 3). Additionally, to replicate the tooth contact during chewing, the contact time between the piston and occlusal surface of the crown was set to 0.2 s with a frequency of 1 Hz (Attiah et al., 2020; El‐Sheikh et al., 2018; Sammour et al., 2019).

After cyclic loading and removal of the crowns, the RTV was measured and recorded using a torque device. Finally, the initial removal torque loss (RTL) percentage, after cyclic loading RTL, and the difference between initial and after cyclic loading RTL were calculated for all abutments (El‐Sheikh et al., 2018). A customized rigid metal mounting jig was used as a holding device to ensure solid fixation during all tightening and un‐tightening procedures (Sammour et al., 2019; Siadat et al., 2015).

$$\mathrm{Removal}\;\mathrm{torque}\;\mathrm{loss}\;\mathrm{ratio}\;\mathrm{before}\;\mathrm{loading}\;\left(\%\;\mathrm{initial}\;\mathrm{RTL}\right)=\frac{\mathrm{Tightening}\;\mathrm{torque}-\mathrm{removal}\;\mathrm{torque}\;\mathrm{before}\;\mathrm{loading}}{\mathrm{Tightening}\;\mathrm{torque}}\times 100$$

$$\mathrm{Removal}\;\mathrm{torque}\;\mathrm{loss}\;\mathrm{ratio}\;\mathrm{after}\;\mathrm{loading}\;(\%\;\mathrm{postload}\;\mathrm{RTL})=\frac{\mathrm{Tightening}\;\mathrm{torque}-\mathrm{removal}\;\mathrm{torque}\;\mathrm{after}\;\mathrm{loading}}{\mathrm{Tightening}\;\mathrm{torque}}\times 100$$

$$\mathrm{Removal}\;\mathrm{torque}\;\mathrm{loss}\;\mathrm{ratio}\;\mathrm{between}\;\mathrm{before}\;\mathrm{and}\;\mathrm{after}\;\mathrm{loading}\;(\%\;\mathrm{difference}\;\mathrm{between}\;\mathrm{initial}\;\mathrm{and}\;\mathrm{postload}\;\mathrm{RTL})=\frac{\mathrm{Removal}\;\mathrm{torque}\;\mathrm{before}\;\mathrm{loading}-\mathrm{removal}\;\mathrm{torque}\;\mathrm{after}\;\mathrm{loading}}{\mathrm{Removal}\;\mathrm{torque}\;\mathrm{befor}\;\mathrm{loading}}\times 100$$

The data were reported as numbers, percentages, and mean ± standard division (SD). The Shapiro–Wilk test was used to assess the normality of the distributions; Levene's test was used to test the homogeneity of variances. Independent *T*‐test and analysis of variance were used to compare the variables. All statistical data were analyzed using IBM SPSS version 24 (IBM SPSS Inc.), and a significance level of less than .05 was considered.

## 3 RESULTS

The initial RTL, RTL percentage after dynamic cyclic loadings, and differences between initial and after cyclic loading RTL were evaluated for each group. Table 1 illustrates the highest RTL in the C10 group, while the lowest RTL percentage was found in the S4 group (before and after loading). The RTL differences ranged from 6.63 ± 0.09 Ncm in the S4 group to 8.22 ± 0.16 Ncm in the C10 group. Table 2 illustrated the impact of abutment type on RTL while keeping the abutment height constant and significant differences were observed in all three groups (*p* < .05). The cast abutments consistently exhibited higher differences between initial and after cyclic lading RTL compared to the stock and milled abutments in all postheights.

**Table 1:** Determine the percentage of removal torque loss (RTL) before and after dynamic cyclic loading and the differences between initial and postloaded RTL for stock, cast, and milled implant abutments.

Groups (*n* = 6 in each)	Initial RTL percentage (Mean ± SD)	Postloaded RTL percentage (Mean ± SD)	RTL differences (Mean ± SD)
Stock 4 mm	25.14 ± 1.08	30.10 ± 1.06	6.63 ± 0.09
Cast 4 mm	35.80 ± 1.15	40.76 ± 1.15	7.73 ± 0.17
Milled 4 mm	31.40 ± 1.94	36.37 ± 1.94	7.24 ± 0.2
Stock 7 mm	31.01 ± 1.57	35.96 ± 1.59	7.17 ± 0.21
Cast 7 mm	37.80 ± 1.27	42.76 ± 1.30	7.79 ± 0.22
Milled 7 mm	34.10 ± 0.71	39.06 ± 0.71	7.2 ± 0.10
Cast 10 mm	39.70 ± 0.86	44.66 ± 0.88	8.22 ± 0.16
Milled 10 mm	37.49 ± 0.62	42.46 ± 0.64	7.93 ± 0.14

Abbreviation: SD, standard deviation.

**Table 2:** The comparison of the removal torque loss (RTL) percentage before and after cyclic loading and the differences between initial and after cyclic loading RTL according to the different heights (4, 7, and 10 mm) for stock, cast, and milled implant abutments.

Height of post	RTL		(Mean ± SD)	*F*	*p* Value
4 mm	Before cyclic loading	Stock (1)	25.14 ± 1.08	110^a^	<.001
		CAST (2)	35.80 ± 1.15		
		Milled (3)	31.40 ± 1.94		
	After cyclic loading	Stock (1)	30.10 ± 1.06	111.22^a^	<.001
		CAST (2)	40.76 ± 1.15		
		Milled (3)	36.37 ± 1.4		
	The differences between initial and after cyclic loading	Stock (1)	6.63 ± 0.09	93.60^a^	<.001
		CAST (2)	7.73 ± 0.17		
		Milled (3)	7.24 ± 0.21		
7 mm	Before cyclic loading	Stock (1)	31.01 ± 1.57	60.46^a^	<.001
		CAST (2)	37.80 ± 1.27		
		Milled (3)	34.10 ± 0.71		
	After cyclic loading	Stock (1)	35.96 ± 1.59	58.60^a^	<.001
		CAST (2)	42.76 ± 1.30		
		Milled (3)	39.06 ± 0.71		
	The differences between initial and after cyclic loading	Stock (1)	7.17 ± 0.21	17.91^b^	<.001
		CAST (2)	7.79 ± 0.22		
		Milled (3)	7.52 ± 0.10		
10 mm	Before cyclic loading	CAST (2)	39.70 ± 0.86	5.92^c^	<.001
		Milled (3)	37.49 ± 0.62		
	After cyclic loading	CAST (2)	44.6 ± 0.88	5.72^c^	<.001
		Milled (3)	42.46 ± 0.64		
	The differences between initial and after cyclic loading	CAST (2)	8.22 ± 0.16	3.81^c^	.002
		Milled (3)	7.93 ± 0.14		

Abbreviation: ANOVA, analysis of variance.

a ANOVA;

b Kruskal–Wallis;

c Independent *T*‐test.

Pairwise comparisons evaluated the differences in the initial RTL, after cyclic loading RTL, and the differences between initial and after cyclic loading RTL among the study groups and demonstrated that all between the studied groups were statistically significant (*p* < .001) for 4 mm. Significant pairwise comparisons were observed among the study groups for 7 mm abutments in relation to the initial RTL percentage (*p* < .001), RTL after cyclic loading (*p* < .001), and the differences between initial and post‐cyclic loading RTL (*p* = .028). However, there was no significant difference in the RTL between stock and milled implant abutments when comparing the initial and post‐cyclic loading values (*p* = .334).

In contrast, no statistically significant differences were observed in the other two comparisons (*p* > .05). On the other hand, pairwise comparisons in the milled abutments group illustrated that after loading in 4‐ and 7‐mm groups, there were no statistically significant differences (*p* = .198) and (*p* = .231), respectively. In contrast, other pairwise comparisons were significant (*p* < .05). The results of evaluating the impact of types and the height of abutments on the RTL percentage before and after loading, and also the differences between initial and after cyclic loading RTL, demonstrated that both parameters (types and height of abutments) significantly influenced the RTL percentage (*p* < .05), (Table 3).

**Table 3:** Evaluation of the impact of types and the height of abutments on the RTL percentage.

RTL	Variable of abutment	Mean Squares	*F* a	*p* **Va**lue
Before cyclic loading	Height	35.152	87.101	<.001
	Type	29.310	47.207	<.001
	Height * Type	32.14	57.9	<.001
After cyclic loading	Height	152	37.100	<.001
	Type	53.310	06.205	<.001
	Height * Type	34.14	47.9	<0.001
The differences between initial and after cyclic loading	Height	87.1	08.66	<0.001
	Type	74.3	91.131	<0.001
	Height * Type	10.0	71.3	0.017

*Note*: * indicate the level of significance is 0.05.

Abbreviations: ANOVA, analysis of variance; RTL, removal torque loss.

^a^ Two‐way ANOVA was conducted to analyze the data.

## 4 DISCUSSION

Due to our findings, the type and height of the abutment have shown a significant impact on the screw loosening in increased CHS. Loosening the abutment screw is an important factor influencing the success and durability of an implant‐based treatment (Hossain et al., 2023; Jayachandran, 2022). According to our findings, the RTL after loading and its difference in stock abutments showed a significant advantage over milled and cast abutments. Also, an abutment with a postheight of 4 mm had a better outcome than the postheight of 7 and 10 mm in maintaining the torque in the screw. On the other hand, in an increased CHS of 12 mm, using a stock abutment with a postheight of 4 mm could effectively minimize screw loosening. Consequently, longer abutments act as force multipliers, mainly when there is an increase in vertical cantilever, resulting in heightened forces applied to the abutment screw (Bulaqi et al., 2015).

Similar to the previous studies (Attiah et al., 2020; El‐Sheikh et al., 2018; Nakano et al., 2021; Sammour et al., 2019), in the present study, to simulate the lateral forces inside the mouth, which have a vital effect on joint stability, a load 5 mm away from the center of the abutment was applied on a flat surface (Attiah et al., 2020; Sakamoto et al., 2016). However, some studies used crowns with an inclination of 30° (Tsuge & Hagiwara, 2009) or applied the force at an angle of 30° to the longitudinal axis (Kim et al., 2012). According to the results, a significant association between the abutment type and the initial and after cyclic loading RTL percentage was observed. Similar to our findings, a study by Ahmed et al. has demonstrated that cyclic loading led to significant screw loosening in both stock and cast abutments. However, they observed that stock abutments exhibited significantly less screw loosening compared to cast abutments, both before and after loading (3.12 Ncm for stock abutments vs. 2.19 Ncm for cast abutments), which was in contrast to our findings (Ahmed et al., 2019). Variations in the applied cyclic loading protocols, including magnitude, frequency, duration, and differences in the assessment methods for screw loosening, such as torque measurement techniques and loosening criteria, could also contribute to the disparities observed.

Another study illustrated that mechanical cycling decreased the torque of both cast and stock abutments. However, there was no significant difference in the torque reduction between the two types of abutments (Junqueira et al., 2013). Furthermore, another study reported no significant differences in the RTV before and after loading among different abutment types (stock, cast, and milled abutments). Therefore, the abutment types did not significantly impact short‐term screw loosening. However, after 10,000 cycles of dynamic loading, the milled abutment did show an effect on initial screw loosening, while the stock abutment and cast abutment did not (Kim & Shin, 2013). On the other hand, Jalalian et al. reported that applying cyclic loading significantly increased vertical misfit, microleakage, and torque loss. Also, they observed no instances of screw loosening in any of the groups (Naseri et al., 2021). In a study by Tartea et al. it has been observed that milled abutments compared to stock abutments, enhance retention, reduce angulation, and decrease the risk of unscrewing or fracturing the screw while increasing the overall size of the abutment (Târtea et al., 2023).

Our results indicated that the S4 and S7 groups showed superior performance compared to M4, M7, C4, and C7 in initial and after cyclic loading RTL, as well as the differences between them. Additionally, among the C10 and M10, the milled abutment exhibited a significant advantage over the cast abutment. These findings highlight the importance of considering the abutment type and postheight in achieving optimal RTL outcomes in dental prosthetic applications. A study by Siadat et al. on screw loosening in stock abutments, comparing CHS of 1.5, 3.5, and 5.5 mm, illustrated no significant difference in initial RTL among the heights. However, the percentage of RTL after loading significantly increased with an increased CHS. The difference in RTV before and after loading may be attributed to surface wear between the screw, fixture, and abutment, minimizing the frictional effect during loading, which initially stabilized the components. The higher vertical cantilever effect and its detrimental impact on screw stability were also mentioned as reasons for the increased percentage of RTL in the 5.5 mm CHS to the 1.5 mm height (Siadat et al., 2015).

Elsayed et al. suggested that increasing the internal hexagon height and decreasing the CHS can increase the marginal fit of the implant‐abutment connection. However, it is essential to note that as the collar height increased, the microgap size also increased, indicating a potential compromise in the stability of the screw joint‐abutment connection. They reported that off‐axis forces can cause the screw threads to settle, decreasing torque and creating vibration movements. As a result, the screw's ability to maintain component stability is diminished, and the gap between the abutment and implant increases (Elsayed et al., 2021). Furthermore, another study reported that the percentage of RTL significantly increased with increased CHS and abutment angle. The percentage of RTV after loading was lower in abutments with a 4 mm collar height compared to those with a 2 mm collar height. They found that the collar height of the abutment acts similarly to a vertical cantilever, thus increasing it intensifies the forces applied to the screw (El‐Sheikh et al., 2018). In their study, the height of the abutment collar was increased, while in the current study, the height of the abutment post was considered a variable. Bordin et al. investigated the impact of abutment collar height and implant length on the biomechanical behavior of morse taper single dental implants with varying crown‐to‐implant ratios with six virtual models, combining different abutment collar heights (short, medium, and long) and implant lengths (11 or 13 mm) through their study. The findings revealed that more extended abutment collars significantly increased stress on the abutment, and screw loosening compared to shorter collars (Bordin et al., 2019).

The current study specifically evaluated the potential benefits of increasing the abutment height in minimizing vertical cantilever and reducing the likelihood of screw loosening. This study is the first to compare abutment heights without modifying their collar height. However, it is important to acknowledge that the experimental in vitro nature of the study represents a limitation, and it is recommended for future studies to investigate the clinical validation to confirm findings, comparative analysis of different abutment designs and materials, long‐term follow‐up evaluations, increased sample size and randomization, and a multidisciplinary approach.

## 5 CONCLUSION

In conclusion, our findings suggest that stock abutments have an advantage over milled and cast abutments regarding RTL after loading. Additionally, a 4 mm postheight abutment is more effective than 7 or 10 mm in maintaining screw torque. In cases with a 12 mm CHS, a stock abutment with a 4 mm postheight may help minimize screw loosening.

## AUTHOR CONTRIBUTIONS

Amirreza Hendi contributed to the conceptualization, data curation, formal analysis, investigation, methodology, project administration, software, visualization, and writing of the original draft. Sobhan Mirzaee contributed to data curation and investigation. Mehran Falahchai contributed to the conceptualization, methodology, supervision, project administration, and writing by reviewing and editing the manuscript.

## CONFLICT OF INTEREST STATEMENT

The authors declare no conflict of interest.

## ETHICS STATEMENT

The study was approved by the ethical committee of the Guilan University of Medical Sciences, Rasht, Iran [IR.GUMS.REC.1400.043].

## Data Availability

The data sets used and analyzed in the current study are available from the corresponding author upon reasonable request.
